# Non-Conscious Effect of Food Odors on Children’s Food Choices Varies by Weight Status

**DOI:** 10.3389/fnut.2017.00016

**Published:** 2017-05-11

**Authors:** Lucile Marty, Héléna Bentivegna, Sophie Nicklaus, Sandrine Monnery-Patris, Stéphanie Chambaron

**Affiliations:** ^1^Centre des Sciences du Goût et de l’Alimentation (CSGA), CNRS, INRA, Univ. Bourgogne Franche-Comté, Dijon, France

**Keywords:** priming, odors, food choices, obesity, children

## Abstract

**Objective:**

Food cues are omnipresent in the daily environment and may influence eating behavior even non-consciously. An increased reactivity to food cues, such as food odors, has been shown to be correlated with obesity in children. The objective of this study is to investigate whether the non-conscious influence of food odors on children’s food choices varies by their weight status.

**Methods:**

Seventy-four children, of whom 29 were obese, took part in this study. The children performed a food choice intention task presented as a computer game in which 30 pairs of food images (a fatty-sweet food picture vs. a fruit picture) successively appeared on the screen. The children had to choose the item “they most wanted to eat at the present moment” for each pair. While performing this task, the children wore a headset in which the microphone foam was odorized with a fruity odor, a fatty-sweet odor or no odor. They performed the intention task three times, one time for each olfactory condition. The odors were non-attentively perceived, i.e., none of the children were aware of the odorization of the microphone foams. The modeled probability is the probability to choose a fruit.

**Results:**

In children with obesity, the fruity odor increased the likelihood of a fruit to be chosen compared to the no-odor condition [OR (95% CL) = 1.42 (1.13–1.78), *P* = 0.0028], while the fatty-sweet odor had no effect on food choice [OR (95% CL) = 1.07 (0.85–1.36), *P* = 0.55]. In children without obesity, both the fruity and the fatty-sweet odors decreased the likelihood to choose a fruit compared to the no-odor condition [OR (95% CL) = 0.76 (0.64–0.90), *P* = 0.0015, for the fruity odor and OR (95% CL) = 0.79 (0.66–0.93), *P* = 0.0062, for the fatty-sweet odor].

**Conclusion:**

The different patterns of results obtained in both groups of children suggest differences in the mental representations activated by non-attentively perceived olfactory cues based on weight status.

## Introduction

Current research in social and cognitive psychology suggests that environmental stimuli can have an effect on information processing and behavior, even when people are not aware of these influences (1, 2). Eating behavior is no exception to this rule. Many cues related to food (e.g., odors, images, and messages) are omnipresent in the daily environment and may affect eating behavior. People are often not even aware of the presence of these cues, and they are even less aware of the impact that such cues can have on their own eating behavior (3–5).

In everyday life, children are surrounded by food cues, whether they are on TV, the internet, or the street in the form of a billboard or a fast-food restaurant. This constant exposure to palatable energy-dense food cues is noted to be one of the factors responsible for the current obesity epidemic (6–8). Despite the fact that children are highly exposed to such food cues, there is a wide variation in their body weight, suggesting different patterns of response to an obesogenic environment. Obesity in childhood requires particular attention considering that children with obesity are more likely to remain obese into adulthood and to develop diabetes and cardiovascular diseases at a younger age (9). Among the food cues that could influence children’s eating behavior, food odors are of particular interest. Indeed, the olfactory bulb has direct connections with the amygdala and hippocampus – two brain areas strongly implicated in emotion and memory (10). This may explain why olfaction is successful at triggering emotions and memories and useful to impact food choice behavior (5). Moreover, from a classical conditioning perspective, the more frequently a cue is associated with a behavior in daily life, the more likely the cue is to prompt this particular behavior. Yet, a food odor is most of the time related to an actual eating situation, more than a visual cue for instance. Children are used to seeing a lot of food pictures outside an eating context, such as on TV, or on the internet, but when they smell food it is usually because someone is cooking for them, or they get in a restaurant or in a bakery where they will actually choose something to eat. Thus, food odors are directly associated with eating situations and then can be viewed as strong predictors of eating behaviors. Moreover, this conditioned association between food odor cues and eating behavior might be stronger in obese than in normal-weight children because they show higher external eating trait, meaning that their eating behavior is more often triggered by food cues (11, 12). Thus, it might be assumed that this strengthened link between food cues and behavior would make food odors more predictive of food choices in obese children. The key point of this study is to focus on the non-conscious influence of food odors on children’s food choices based on their weight status.

To our knowledge, only two experimental studies have compared the responsiveness for attentively perceived food odors in children based on their weight status (13, 14). In one study, obese children between 8 and 12 years consumed more palatable high-calorie foods than their lean counterparts after attentively smelling them (14), while another study found that 6- to 11-year-old children with obesity showed increased lip sucking in response to an attentive exposure to both high-energy food pictures and food odors (13). Thus, attentively perceived food odors seem to differentially affect children’s eating behavior based on their weight status. However, the influence of non-attentively perceived food odors on children eating-related behavior based on weight status has never been investigated. Yet, there are strong evidences in the literature that an eating behavior can also be triggered by non-attentively perceived food cues (3, 4) and especially food odors (5). Thus, investigating the influence of non-attentively perceived food odors in children with and without obesity could help to better understand children’s eating behavior and to highlight potential psychological mechanisms underlying eating behavior that may be associated with early obesity. To explore this question, an olfactory priming paradigm was adapted to children.

The priming paradigm initially developed in cognitive psychology (15) consists of two phases. In the first phase, participants are incidentally exposed to a stimulus called a “prime,” which can belong to any sensory modality (e.g., visual, auditory, olfactory). During the exposure, mental representations related to the prime are activated (16, 17). In the second phase, the unconscious effects of the activation are evaluated using indirect memory tasks (18) in which no reference is made to the prior phase. Priming effects require the participant to be unaware of the influence of the primes on later tasks (1). Primes can automatically activate associated representations in memory, leading to higher accessibility. This accessibility then spreads to related constructs via an associative network (19, 20). In line with spreading-activation theory, priming a given construct in memory leads to the spontaneous activation of related constructs in memory. In a precursor study on olfactory priming, Holland and colleagues explored the influence of a non-attentively perceived scent of citrus, typical of all-purpose cleaners, on thinking and behavior in adults (21). The results showed that the presence of citrus scent enhanced the accessibility of the behavior concept of cleaning, which was indicated by the faster identification of cleaning-related words in a lexical-decision task as well as a higher frequency of listing cleaning-related activities when describing expected behavior during the day. Moreover, it was established that exposure to the citrus scent also influenced actual performance of cleaning behavior. It is important to note that the participants were not aware of the presence of the scent or the fact that their cognition and behavior were affected by the scent. Thus, a non-attentively perceived odor (e.g., a citrus scent) can non-consciously activate a concept (e.g., the cleaning concept) that may subsequently guide behavior in adults.

In the food domain, a recent study on olfactory priming was conducted with adult participants. The study demonstrated that a non-attentively perceived odor of melon led to a faster identification of the word “melon” during a lexical-decision task and that participants who were exposed to the melon odor were more likely to choose appetizers with vegetables in a task that involved choosing from a menu (22). Two other studies investigated the influence of non-attentively perceived food odors on real food choices in adults which have shown that a pear odor increased the proportion of choices of a fruity dessert (an apple purée) whereas a chocolate-croissant odor increased the proportion of choices of a fatty-sweet dessert (a waffle) (23, 24). The authors of these studies hypothesized that a given food odor, even non-attentively perceived, activated an odor-congruent concept (i.e., a fruity odor activated the fruit and vegetables concept, while a fatty-sweet odor activated the fatty-sweet foods concept) resulting in the facilitation of odor-congruent food choices.

The investigation of fruity and fatty-sweet odors and their influence on eating behavior is particularly relevant in terms of public health. Fruit and fatty-sweet foods are distinct regarding their typical sensory properties (e.g., taste, aroma, etc.), but they are also opposite regarding their healthiness properties. Thus, in the context of increasing childhood obesity, it is of particular interest to investigate the unconscious influence of fruity versus fatty-sweet food odors on children’s food choices based on their weight status. Indeed, this investigation could give insights into the psychological mechanisms that underlie obesity in children.

The main objective of the present study was to determine whether olfactory primes (a fruity and a fatty-sweet food odor) differentially influence children’s food choices based on their weight status. To do so, a food choice intention task was developed. It was presented as a computer game in which children must choose “what they most want to eat at this moment” by selecting a food among pairs of pictures (a fruit vs. a fatty-sweet food). This study is the first to develop such methodology tailored for children. Considering the existing literature on the influence of attentively perceived food odors in children and the influence of non-attentively perceived food odors in adults, the most relevant hypothesis is that a fruity odor may guide children’s food choices toward more fruit, whereas a fatty-sweet odor may guide food choices toward more fatty-sweet products. Moreover, an amplified effect of the olfactory primes in children with obesity, compared to children without obesity, is expected (13, 14), resulting in a larger increase in the likelihood to choose a fruit when exposed to the fruity odor and also the likelihood to choose a fatty-sweet product when exposed to the fatty-sweet food odor.

## Materials and Methods

### Participants and Design

The children included in this study were between 6 and 11 years old, attended an elementary school, and had no food allergies. They were recruited from a population registered in the Chemosens Platform’s PanelSens database. This database complies with national data protection rules and has been vetted by the appropriate authority (Commission Nationale Informatique et Libertés – CNIL – no. 1148039). Children with obesity were specifically recruited from pediatric weight care consultations at the Dijon public hospital. The study was conducted in accordance with the Declaration of Helsinki and was approved by the local ethical committee (Comité pour la Protection des Personnes EST-1 Burgundy, file number: 2015-A01547-42). Written informed consent was obtained from parents before their child’s participation in the study. All applicable institutional and governmental regulations concerning the ethical use of human volunteers were adhered to during this research study. In return for their participation, the parents received a €10 voucher.

The children and their parents were invited to a 1-h session during after-school snack time (5 p.m. to 6 p.m.). Parents were informed that their child would be served a snack in the laboratory and were asked not to offer their child a snack during the afternoon prior to the experiment. Children were tested in small groups (maximum of eight children) in individual booths in the laboratory. During the experimental session, children performed a food choice intention task on a computer while they were incidentally exposed to food odors. The food choice intention task was presented as a game in which 30 pairs of food pictures (fruit vs. fatty-sweet food pictures) successively appeared on the screen. During the task, the children wore a headset (Samar, London, UK) to listen to the instructions. The foam on the headset’s microphone was odorized with either the aroma of a pear or pound cake, along with non-odorized foam that would serve as the control condition. This odorization method was inspired by the work of Leleu et al. (25). A double-blind procedure was used for odor exposure. First, to ensure that the participants would not be attentive to the olfactory cues during the experimental session, they were given a false purpose to the study, namely that the experiment was designed to examine how children process visual information. Second, the researchers who provided the headsets to the children did not know which odorant was present on the foams of the microphones. Each child performed the food choice intention task three times, one time for each olfactory condition (i.e., they saw 30 pairs of foods in each olfactory condition). Thus, the impact of the olfactory condition on food choices was tested in a within-subject design. This is the first attempt to develop a within-subject olfactory priming methodology, thus it was impossible to perform an *a priori* power calculation in order to determine the sample size needed to observe the expected effects. Nonetheless, a recent study investigated the influence of odors (but attentively perceived) on recognition of facial expressions using a within-subject design (25). The authors recruited 31 adults and saw an effect of odors on the outcome variable. In line with this study, we aimed at recruiting about 30 children in each group (with and without obesity).

The choice of the olfactory primes and the characteristics of the food pictures for the food choices intention task were determined after preliminary studies that are detailed in the following sections. The children involved in these preliminary experiments were different from those involved in the priming experiment, but they shared similar characteristics (age, gender, and weight status).

### Olfactory Primes

There were two methodological challenges regarding the olfactory primes, the aroma selection, and the appropriate concentration chosen for each odor. The pear and pound cake food aromas sold by Meilleur du Chef© (Bassussarry, France) were selected on the basis of a preliminary study that aimed to identify fruity and fatty-sweet odors well identified and liked by children. Four fruity odors (pear, wild strawberry, melon, and apple) and four fatty-sweet odors (pound cake, marshmallow, almond, and cotton candy) were initially tested with 15 children. For each odorant, they were asked to (1) rate their liking for the odor on a continuous scale (from “I don’t like it at all” to “I like it very much” coded from 0 to 10), (2) describe how they felt like while smelling the odor, (3) identify the odor, and (4) identify the odor by means of a four-alternative forced-choice identification task (Table 1). Concerning the fruity odors, melon, wild strawberry, and pear appeared to be good candidates as they were similarly appetizing and enjoyed and highly identified as fruity odors by the children in the study. The pear aroma was selected on the basis of previous work in adults (22, 24). Concerning the fatty-sweet odor, the pound cake aroma was selected because it was the most appetizing for the children, and it was also properly identified as a fatty-sweet odor by 70% of the children.

**Table 1:** **Results of the pretests to select the food olfactory primes (*n* = 15)**.

	Liking	Feel like eating (%)	Correct free identification^a^ (%)	Correct forced-choice identification^a^ (%)
**Fruity odors**				
Pear	7.4 ± 2.9	44	50	67
Wild strawberry	8.6 ± 1.8	56	71	89
Melon	7.4 ± 3.5	50	33	100
Apple	9.1 ± 1.0	56	50	44
**Fatty-sweet odors**				
Pound cake	7.4 ± 3.5	70	70	70
Marshmallow	8.4 ± 2.1	22	0	89
Cotton candy	7.5 ± 2.5	22	43	100
Almond	7.2 ± 2.6	33	43	67

*^a^Identification is considered as correct when a fruity odor (resp. a fatty-sweet odor) is indeed identified as a fruit odor (resp. a fatty-sweet food odor)*.

To expose children to these two different odorants, headset microphone foams were odorized (25). The odorization procedure was designed to (1) obtain stable odor intensity during the experiment and (2) obtain very low odor intensity such that children would not attentively perceive the odor. One hour before each experiment, the two odor solutions (15 µL) were absorbed into the polyurethane foam of the microphones of two distinct sets of headphones. The pear aroma was undiluted, and the pound cake aroma was diluted in propylene glycol to reach a concentration of 10^−1^ v/v. The control headsets with non-odorized microphone foam were indistinguishable from the other headsets. The subjective intensity equivalence of both odorants was evaluated by six adult raters. To ensure that the odorant application process resulted in a non-attentively perceived odor intensity, 12 children participated in a rehearsal of the entire study protocol. They were successively exposed to the three olfactory contexts (control, pear, and pound cake) in a counter-balanced order. They were then asked by a researcher to report orally what they thought about the headset and whether they noticed anything unusual with it. Only one of them had noticed the presence of an odor. Moreover, all of them were able to detect an odor when the researcher asked questions that focused their attention to it during a post test.

### Food Choice Intention Task

This task was presented as a computer game in which 30 pairs of food pictures (fruit vs. fatty-sweet food pictures) successively appeared on the computer screen. The task included a familiarization phase during which children performed three training trials. One picture appeared on the left side of the screen and another on the right simultaneously. Children had to choose “what they most wanted to eat at this moment” by selecting the appropriate food picture. They answered by pressing an identified key on the left or right side of the keyboard. For each pair of test pictures, the answer (coded 0 if the fatty-sweet food was chosen; 1 if the fruit was chosen) and the reaction time (ms) were recorded.

Pictures used for this task came from the Full4Health standardized food images database (26). The food pictures, presented as a pair, had to (1) display a fatty-sweet food and a fruit, (2) be properly identified by children, (3) be contrasted in healthiness perceived by children, and (4) be similarly liked by children. Sixty-three pictures of sweet foods (divided into two batches) were pre-tested with 61 children who were recruited in a holiday center for school children. Each child was presented with one batch of food pictures by a researcher during a face-to-face interview. For each food picture, children were asked to (1) identify the food presented, (2) rate their liking for this food on a continuous scale (from “I don’t like it at all” to “I like it very much,” coded from 0 to 10), and (3) rate their perception of healthiness of this food on a continuous scale (from “It is not healthy at all” to “It is very healthy,” coded from 0 to 10). Only 43 food pictures were properly identified by at least 80% of the children and subsequently used for this study. The food choice intention task was programmed to randomly draw with replacement 30 pairs of one fruit picture and one fatty-sweet food picture among the 348 pairs that met the criteria of having a similar liking score (no more than a 2-point difference between the two foods presented together) and contrasted perceived healthiness (more than a 4-point difference between the two foods presented together). This means that each time the task was run (i.e., three times per child, one time for each olfactory condition) probably led to a unique combination of 30 food pairs.

### Procedure

The children entered the experiment room with the researchers while their parents remained in the waiting room. Before beginning the experiment, the content of the session was explained to the children. A researcher explained that they would be equipped with a headset for the game instructions and that they could not touch or remove the headset.

Each child was seated in an individual booth in front of a computer and equipped with the first headset (olfactory condition 1) installed by a researcher. The child then performed the first food choice intention task. When finished, a researcher removed the first headset and gave the child five boards of “Where’s Waldo?” This part of the experiment was presented to the children as a new game but it was simply a distraction in order to exchange the first headset (olfactory condition 1) with the second (olfactory condition 2). After precisely 10 min, the boards were recovered and the child was equipped with the second headset (olfactory condition 2). The child performed the food choice intention task again, and this procedure was replicated a third time for olfactory condition 3.

At the end of the priming session, children answered a debriefing questionnaire designed to determine whether they had or had not suspected the real goal of the study. In particular, it aimed to confirm that children had not attentively perceived or identified the prime. During face-to-face interviews, all the children were asked by a researcher to report what they thought about the whole experiment, what they thought about the headset, and whether they noticed anything unusual about it.

Before leaving, trained researchers measured the children (weight and height). Weight (kilograms) was measured with the child wearing light clothes and no shoes to the nearest 0.1 kg using a digital scale (Soehnle, Benfeld, Germany); height (centimeters) was measured to the nearest 0.1 cm with the child in a standing position without shoes using a stadiometer (Seca Leicester, Birmingham, UK). Body mass index (BMI) was calculated and transformed into age- and sex-standardized *z*-scores (*z*-BMI) based on the French reference data (27). Children were considered obese if *z*-BMI ≥2.

### Statistical Analysis

The three olfactory conditions were presented in a counter-balanced order. The nature of food choices was coded as 0 when the fatty-sweet food was chosen or as 1 when fruit was chosen. The olfactory condition effect (pear odor, pound cake odor, and no odor; within subjects), the weight status effect (normal-weight vs. obese, between subjects), and the interaction between olfactory condition and weight status on the nature of the food choices were tested. A generalized linear model with a binomial distribution and logit link was applied to the nature of food choices. For the olfactory condition effect, the no-odor condition was taken as reference in the model (i.e., only the two contrasts pear odor vs. no odor and pound cake odor vs. no odor were tested). Individual reaction times were transformed using a 1/*Y* transformation into reaction speed to improve the symmetry of the distribution as in Gaillet et al. research (22). The olfactory condition effect, the weight status effect and the nature of the food choices effect as well as all their interactions on reaction speed were tested. A generalized linear model with a normal distribution was applied to the reaction speed. In these two models, the child effect was considered as random, and these models were adjusted for sex and age of the children and for the order of presentation of the primes.

These models were estimated with the SAS GLIMMIX procedure from the SAS software version 9.3 (SAS Institute, Inc., 2012 SAS^®^ 9.3., Cary, NC, USA). Significance was set at *P* = 0.05. When no precision is given, the results are expressed as the means ± SD.

## Results

### Sample

Seventy-five children took part in the experiment. One participant had cold and asked to blow his or her nose during the experimental session. These data were removed from the analyses. Data from the debriefing questionnaire showed that the children were not aware of the actual goal of the study (i.e., to study the effect of olfactory priming on food choices) and that none of them had noticed the presence of an odor on the microphone foam. Consequently, none of the participants were excluded on this basis. Thus, analyses were run on a sample of 74 children; 45 of whom were normal weight, and 29 were obese with similar characteristics in terms of age and sex (Table 2).

**Table 2:** **Participants’ characteristics**.

	All (*n* = 74)	Children without obesity (*n* = 45)	Children with obesity (*n* = 29)	*P*
Age^a^	8.7 ± 1.6	8.6 ± 1.6	9.0 ± 1.5	0.23^b^
Sex (% girls)	46	49	41	0.52^c^
*z*-BMI^a^	1.44 ± 1.92	0.13 ± 1.05	3.46 ± 0.97	<0.0001^b^

*z-BMI, z-score of body mass index*.

*^a^Values are means ± SD*.

*^b^T-test*.

*^c^Chi-square test*.

### Food Choices

**Figure 1:**
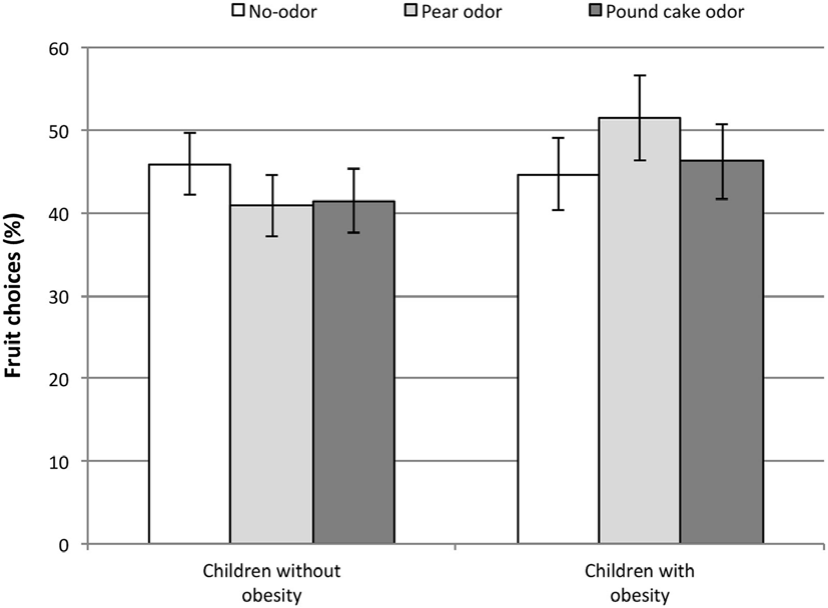
**Percentage of fruit choices in the food choice intention task among normal weight (*n* = 45) and obese children (*n* = 29) in each olfactory condition (values are means ± SEM)**.

On average, the number of fruit chosen was 13.4 ± 7.6 over the 30 pairs of pictures of the food choice intention task (Q1 = 7; median = 13; Q3 = 18). See Figure 1 for details.

The olfactory condition effect, the weight status effect, and the interaction between olfactory condition and weight status on the nature of the food choices were tested. Type III tests of the fixed effects revealed that the main effects of olfactory condition and weight status were non-significant [*F*(2, 6,580) = 1.48, *P* = 0.23 and *F*(1, 6,580) = 0.79, *P* = 0.39, respectively], but the interaction between the olfactory condition and weight status was significant [*F*(2, 6,580) = 11.72, *P* < 0.0001] which demonstrated a differential impact of the olfactory condition on the nature of food choices based on the child’s weight status. Analyses were stratified on weight status to describe this differential effect. The results are presented Table 3. In normal-weight children, the pear odor as well as the pound cake odor significantly decreased the probability of choosing a fruit comparatively to the control condition. In children with obesity, the pear odor increased the probability of choosing a fruit comparatively to the control condition, but the pound cake odor had no significant effect on food choices.

**Table 3:** **Effects of the olfactory condition on the nature of food choices: weight status-stratified analysis**.

	Children without obesity (*n* = 45)		Children with obesity (*n* = 29)	
	OR (95% CL)^a^	*P*	OR (95% CL)^a^	*P*
Pear odor vs. no-odor	0.76 (0.64-0.90)	0.0015	1.42 (1.13-1.78)	0.0028
Pound cake odor vs. no-odor	0.79 (0.66-0.93)	0.0062	1.07 (0.85-1.36)	0.55

*^a^The nature of food choices was coded as 0 when a fatty-sweet food was chosen or as 1 when a fruit was chosen: the tested probability in these models is food choice = 1*.

### Reaction Time

On average, the reaction time was 3,547 ± 4,268 ms (Q1 = 2,220, median = 2,830, Q3 = 3,765). The olfactory condition effect, the weight status effect, and the nature of the food choices effect as well as all their interactions on reaction speed were tested. Type III tests of the fixed effects revealed that the main effects of olfactory condition, weight status, and nature of food choices on the reaction speed were non-significant [*F*(2, 6,573) = 1.45, *P* = 0.23; *F*(1, 6,573) = 0.02, *P* = 0.88 and *F*(1, 6,573) = 1.16, *P* = 0.28, respectively]. Moreover, none of the interactions were significant (olfactory condition × nature of the food choices: *F*(2, 6,573) = 0.04, *P* = 0.96; nature of the food choices × weight status: *F*(1, 6,573) = 1.45, *P* = 0.23; weight status × olfactory condition: *F*(2, 6,573) = 1.18, *P* = 0.31; olfactory condition × nature of the food choices × weight status: *F*(2, 6,573) = 1.69, *P* = 0.19). These results indicated that the reaction speed was not modified by the olfactory condition, by the weight status of the children nor by the nature of food choices.

## Discussion

The present research aimed at exploring the non-conscious influence of food odors on children’s food choices. The results highlighted a differential impact of non-attentively perceived odors depending both on the nature of the odors (fruity vs. fatty-sweet) and on children’s weight status (with vs. without obesity). Whereas fruity and fatty-sweet odors increased the likelihood to choose fatty-sweet foods in normal-weight children, a fruity odor increased the likelihood to choose fruit in children with obesity. This pattern of results is different from our initial hypotheses but can be explained considering the differences in the mental representations of food between children according to their weight status.

Holland and colleagues have shown an enhanced accessibility to the concept of cleaning as a result of exposure to the odor of a typical cleaner, which influences the actual performance of cleaning behavior in adults (21). Based on this demonstration and on previous results regarding the effect of olfactory priming on food choice in adults (22–24), the hypothesis of this study was that a fruity or a fatty-sweet odor would enhance the accessibility of the fruit or fatty-sweet foods concept and would increase fruit or fatty-sweet food choice, respectively. Surprisingly, both a fruity and a fatty-sweet odor increased the likelihood to choose fatty-sweet foods in normal-weight children, whereas the same fruity odor increased the likelihood of choosing fruit in children who were obese. We suggest two possible interpretations for these results, as follows: (1) a sensory explanation: normal-weight children, unlike children with obesity, were not able to accurately differentiate the two food odors; and (2) a cognitive explanation: the fruity and the fatty-sweet odors activated the same mental representation in normal-weight children, leading to an increase in fatty-sweet food choices, whereas the fruity odor activated a different mental representation in children with obesity, leading to an increase in fruit choices.

Considering the first interpretation, the normal-weight children may not have been able to distinguish between the fruity and the fatty components of the two sweet odors. This hypothesis is unlikely given the research and care that was put into choosing the odors. Previous studies comparing olfactory sensitivity between normal-weight and obese subjects led to conflicting results. Some studies reported a decrease in olfactory sensitivity with increasing BMI in adults (28–30), whereas another study in children reported an increase in olfactory sensitivity with increasing BMI (31). Moreover, recent studies have shown that adults with obesity were better at detecting food odors than adults without obesity (32, 33). In summary, there is no strong evidence of a difference in the ability to properly identify food odors according to a subject’s weight status, whether the subjects are adults or children.

The second interpretation is based on a previous study showing that children have distinct attitudes toward food based on their weight status (34, 35). While children without obesity primarily categorized food items according to affective criteria (“This food is yummy”/“This food is yucky”), children with obesity rather used cognitive criteria (“This food makes me strong”/“This food makes me fat”). Children without obesity have dominant pleasure-based attitudes toward food, and children with obesity have dominant nutritional-based attitudes. In a study involving adults, healthy eating prime words were subtly placed in the context of a “recipe flyer” handed out to shoppers when they entered a store, and its effect was assessed after the shopper had checked out by examining their cash register receipts for snack-item purchases (36). The authors reported less unhealthy snack food purchased when healthy eating primes were present but only for obese shoppers for whom the dieting goal was more important compared to non-obese shoppers. This demonstrates that behavioral priming effects depend on the relevance of the prime to the person’s goals. Indeed, a recent meta-analysis showed that the behavioral priming effect was significantly stronger when the goal being primed was of personal importance to the participant (37). Thus, the same prime could enhance the accessibility of different concepts according to the internal representation retrieved by the prime and consequently have distinct effects on behavior. A food odor may enhance the accessibility of distinct mental representations associated with the food (e.g., pleasure, nutritional considerations, and dieting goals) according to individual characteristics, such as attitudes toward food. In the present study, it may be assumed that the fruity and fatty-sweet odors would have enhanced the accessibility of the food pleasure concept in children who were normal-weight, for whom it is the concept most closely associated with food in general (35). Consequently, it would have guided them toward more fatty-sweet food choices. Indeed, fatty-sweet foods are more commonly linked with palatability and food pleasure than fruit (e.g., palatable foods are associated with festive situations). On the contrary, a fruity odor would have enhanced the accessibility of nutritional considerations in children with obesity due to the dominance of nutritional-based attitudes (34), resulting in more fruit choices, which can be considered as more frequent healthy food choices. The reasons why the fatty-sweet odor did not influence the food choices of the children with obesity need to be considered for future research. Indeed, the expected increase in fatty-sweet food choices when exposed to a fatty-sweet odor was not observed in children with obesity. Counteractive-control theory has been proposed to account for the activation of self-control processes in the face of temptation (38). In line with this theory, Coelho et al. have shown that food odor-exposed adults restrained eaters ate less than did non-exposed restrained eaters (39). Yet, restrained eating was measured in this study by using the restrained eating scale of the Dutch Eating Behavior Questionnaire for Children (40), and the results confirmed that the obese children were significantly more restrained than the other children (restrained eating score in obese children: 3.1 ± 0.75, in normal-weight children: 2.6 ± 0.85, *T*-test *P* = 0.02). Thus, more frequent healthy food choices in children with obesity when exposed to a palatable food odor could have also been expected. Therefore, the lack of a significant influence of the fatty-sweet food odor on children with obesity does not necessarily mean that they were not influenced by this odor, but it may reflect discrepant goals regarding palatable foods (achieving pleasure vs. achieving dieting goals).

Olfactory-congruent food choices (i.e., fruit choice with the fruity odor and fatty-sweet product choice with the fatty-sweet odor) may have been expected to be faster than olfactory-non-congruent food choices. However, in the task presented here, children were not asked to perform the task as quickly as possible, which led to a high variability in reaction times. A child could make a choice quickly or take time to consider the pros and cons for each pair of pictures. Reaction times are commonly used to reflect automatic and spontaneous activation (41) but might not constitute a relevant variable for a task that requires a deliberative decision, which may be the case here.

The food choice intention task measured hypothetical food choices with successive two-option forced-choice alternatives and no actual food choices were measured; this can be considered as a potential limitation. This questions the generalizability of our findings in a real food choice setting with more than two options. Nonetheless, a previous study in adults has shown that a non-attentively perceived fruity odor could influence food choices from a menu including 10 choices for each course category of a typical French meal (starter, main course, and dessert) (22). Moreover, regarding the comparison of hypothetical food choices and real food choices, the existing literature on adults suggests that food choice intentions would be good predictors of actual food choices (22, 24). It suggests that the findings of the present study should be replicable for real food choices in a situation including more than two food options. However, further research in real food choice setting – such as an appealing snack food buffet that would offer a range of sweet foods to the children for their after-school snack, for instance – is needed to confirm and extend our results.

Our findings raised some hypotheses that need to get addressed in future research to better understand the differences between obese and normal-weight children regarding their food odor reactivity. First; our results suggest that the accuracy in odor differentiation at low concentration levels might vary by children’s weight status. It would be interesting to test this hypothesis in future research by developing a child-adapted methodology to compare food odor identification and discrimination as well as olfactory thresholds between obese and normal-weight children. The “Sniffin’ Sticks” methodology developed by Hummel could be a child friendly way to asses such differences (42, 43). Second, we suggested that the different patterns of results obtained in both weight status groups of children might be due to differences in the mental representations activated by non-attentively perceived food odors. We hypothesized that the pear and the pound cake odors might activate the food pleasure concept in normal-weight children, whereas the pear odor might activate the nutrition concept in obese children. To test this hypothesis, the activated concepts by food odors have to be elucidated. Holland and colleagues showed that the presence of citrus scent enhanced the accessibility of the behavior concept of cleaning using a lexical-decision task (21). Participants were asked to indicate as quickly and accurately as possible whether a letter string appearing on a computer screen was an existing word, and participants in the scent condition responded faster to cleaning-related words than did participants in the control condition (21). Thus, one trail to test our hypothesis could be to ask children with and without obesity – as long as they can read – to perform a lexical-decision task using related words to the concepts of food pleasure vs. nutrition while they are exposed to the pear odor, the pound cake odor, or no-odor. Faster answers to food pleasure- or nutrition-related words in presence of a food odor would indicate the activation of the related concept by the odor.

In conclusion, a major difference was found between normal-weight children, for whom fruity and fatty-sweet food odors exposure resulted in more fatty-sweet food choices, and children with obesity, for whom fruity food odor exposure resulted in more frequent healthy food choices. Olfactory priming did not influence the children in the same manner, perhaps due to personal representations activated by the food odor, even if further research is needed to clarify this point. The priming paradigm allows us to access implicit mechanisms underlying food choice. These results help to better understand the implicit relationship between children’s eating behavior and food cues in their environment based on weight status, avoiding the social desirability bias of explicit investigation methods. The primed concept, and the consecutive food choices, depends both on the nature of the prime itself and on the personal representation linked to the prime. Priming as an interventional tool in public health must be carefully controlled, with consideration of the personal goals of the children to avoid counter-productive effects.

## Ethics Statement

This study was carried out in accordance with the recommendations of the Comité pour la Protection des Personnes EST-1 Burgundy with written informed consent from all subjects. All subjects gave written informed consent in accordance with the Declaration of Helsinki. The protocol was approved by the Comité pour la Protection des Personnes EST-1 Burgundy, file number: 2015-A01547-42.

## Author Contributions

LM designed the study, collected data, completed the statistical analysis, and drafted the manuscript. HB collected data. SC designed the study and contributed to the writing of the manuscript. All the authors helped interpret the findings, made a critical revision of the manuscript, read and approved the final manuscript.

## Conflict of Interest Statement

The authors declare that the research was conducted in the absence of any commercial or financial relationships that could be construed as a potential conflict of interest.
